# Dual blockade of adenosine A_2A_ and A_2B_ receptors is required to reverse NECA-induced immunosuppression in human macrophages: Implications for cancer immunotherapy

**DOI:** 10.3389/fimmu.2026.1845291

**Published:** 2026-09-14

**Authors:** Foteini Patera, Meryem Gultekin, Anna Malecka, Bryony Heath, Tajkia Musarrat, Aanchal Preet Kaur, Poulam M. Patel, Stephen J. Hill, Andrew M. Jackson, Hester A. Franks

**Affiliations:** 1 Centre for Cancer Sciences, Biodiscovery Institute, University of Nottingham, Science Road, University Park, Nottingham, United Kingdom; 2 University of Nottingham and University of Birmingham Centre of Membrane Proteins and Receptors, The Midlands, United Kingdom; 3 School of Veterinary Medicine and Science, University of Nottingham, Nottingham, United Kingdom; 4 Division of Physiology, Pharmacology and Neuroscience. School of Life Sciences, Medical School, University of Nottingham, Nottingham, United Kingdom

**Keywords:** adenosine, cancer immunotherapy, human, macrophage, NECA, receptor

## Abstract

Adenosine accumulation within the tumour microenvironment is a major barrier to effective cancer immunotherapy, driving potent immunosuppression through adenosine receptor signalling. While current clinical strategies primarily target the A_2A_ adenosine receptor, emerging evidence suggests that adenosine-mediated immune regulation, particularly in human myeloid cells such as macrophages, involves coordinated signalling through both A_2A_ and A_2B_ receptors. In this study, we define for the first time the adenosine receptor pathways responsible for the immunosuppressive effects of the adenosine analogue 5′-N-Ethylcarboxamidoadenosine (NECA) in human monocyte-derived macrophages and determine the downstream consequences for T cell function. NECA-conditioned macrophages acquired an immunosuppressive, M2-like phenotype, characterised by reduced production of pro-inflammatory cytokines (IL-12, IL-23, IL-6, TNFα) and increased expression of VEGF-A and IL-10. Functionally, these macrophages impaired T cell effector responses, markedly reducing Th1-type responses as indicated by ablation of IFNγ production. Importantly, reversal of macrophage polarisation and restoration of T cell function could be achieved by simultaneous blockade of both A_2A_ and A_2B_ receptors. These findings identify the potential of simultaneously inhibiting the A_2A_ and A_2B_ receptors as a novel key strategy for overcoming adenosine-driven immunosuppression and enhancing the efficacy of cancer immunotherapies targeting the tumour microenvironment.

## 1 Introduction

Cancer immunotherapy has revolutionised management of some solid cancers, most notably metastatic melanoma. The most common immunotherapy in clinical practice is checkpoint blockade, involving monoclonal antibodies directed at the CTLA-4 and PD-1/PD-L1 receptors. Single (anti-PD-1) or dual (anti-PD-1 plus anti CTLA-4) immunotherapy for metastatic melanoma has increased median survival from 6 months in the chemotherapy era to over 5 years, with 52% 10 year melanoma-specific survival following dual immunotherapy (1). More recently, LAG3 targeting has also shown benefit in metastatic melanoma (2). These therapies enhance T cell activation, allowing immune attack of tumours. However, this only works in a limited proportion of cancers and patients. The tumour microenvironment (TME) itself is a key target to improve immunotherapy; altering this environment to support immune attack is a priority. Macrophages are an important mediator and determine whether the TME supports or suppresses anti-tumour immunity (3), with macrophages known to impact T cell function. Classically, macrophages have been considered as ‘M1’ (pro-inflammatory, anti-tumour, capable of producing high levels of pro-inflammatory IL-12) and ‘M2’ (anti-inflammatory, pro-angiogenic, tumour-supporting, capable of producing high levels of anti-inflammatory IL-10). In the TME, they are referred to as ‘tumour-associated macrophages’ (TAMs), are typically more ‘M2’-type and are detrimental to anti-cancer immune responses, producing anti-inflammatory, pro-tumour factors such as IL-10 and VEGF. Repolarising these TAMs to a more immunogenic, anti-tumour phenotype would support immunotherapeutic approaches.

Adenosine has been shown to be immunosuppressive, and can be present at higher levels in the TME than in physiologically normal tissues (4, 5). Adenosine in the extracellular space is generated from ATP by the ectoenzymes CD39 and CD73, present on many cells in the TME including malignant cells, immune cells and stromal cells (6). A non-canonical mechanism of adenosine generation also exists involving NAD+ which is converted to adenosine via CD38, CD203a and CD73 (7). Levels of CD73 (the terminal common enzyme for adenosine generation) are prognostic in a range of cancers (8). ‘Adenosine high’ gene signatures have been defined and demonstrated either poor prognosis in tumours with this signature or responsiveness to adenosine-targeting therapies associated with this signature (9–11). Pre-clinical models have shown that anti-PD-1 therapy is less effective in CD73hi tumours, and that anti-PD-1 therapy induces upregulation of adenosine receptors (12).

Adenosine has a short half-life and is rapidly synthesised and degraded in tissues. For *in vitro* studies, where maintenance of steady levels is challenging due to rapid breakdown of exogenous adenosine, the more stable adenosine analogue 5’-N-Ethylcarboxamidoadenosine (NECA) is commonly used instead. Adenosine and NECA act via four G-protein coupled receptors: A_1_, A_2A_, A_2B_ and A_3_, which are variably expressed on different cell types in the TME (13). A_2A_ is a high-affinity receptor receptor (EC50 <1µM) (14). In contrast, A_2B_ has lower affinity with an EC50 of 24µM (14). Therefore, whilst A_2B_ may have a minimal role in healthy tissues with low levels of adenosine, it becomes significant in tumours with their higher levels of adenosine. In this regard, adenosine concentrations in the TME reach levels well above the EC50 of the A_2B_ receptor (5). In T cells, adenosine predominantly mediates its immunomodulatory effects through A_2A_ (15–17). Signalling through A_2A_ suppresses effector T cell function, increases regulatory T cell activity (16) and impairs cytotoxic cytokine secretion (17).

Multiple adenosine-targeting agents are now in early-phase clinical trials for cancer, often as a combination approach (18). Most of these target either the A_2A_ receptor or CD73. Some studies have shown encouraging results, including a small-molecule A_2A_ inhibitor +/- Atezolizumab in renal cancer, demonstrating some partial responses in heavily pre-treated patients (9) and another study demonstrating benefit of adding anti-CD73 treatment to Durvalumab after concurrent chemo-radiotherapy for non-small cell lung cancer (19). Others have started reporting with acceptable safety profiles and some indications of tumour response in both heavily pre-treated patients and in tumour types which are typically immunotherapy resistant (20, 21). However, further publications show disappointing efficacy of A_2A_ or adenosine-generation targeting strategies (22–25). Taken together, these early results indicate that it may be necessary to block multiple adenosine receptors to prevent adenosine-mediated immune suppression in the TME.

Data from murine systems shows that adenosine suppresses production of key macrophage immune-activating cytokines including IL-12 and TNFα but that the A_2A_ receptor alone does not mediate this effect (26). Furthermore, a switch towards an anti-inflammatory phenotype was observed in human monocytes treated with adenosine (decreased IL-12/TNFα, increased IL-10) (27, 28). These cytokines are key to anti-tumour immune attack by T cells. In human monocyte-derived macrophages, adenosine increases VEGF production (29). Importantly, in some tumour types macrophages appear to mediate the effect of adenosine more potently than T cells (11). Gene expression data is variable but overall demonstrates that both the A_2A_ and A_2B_ receptors are expressed in myeloid cells (30–32). Reliably quantifying protein-level expression of adenosine receptors is challenging. Due to the small extracellular part of G-protein-coupled receptors, it is notoriously difficult to generate high-affinity and specific antibodies to these receptors, resulting in a lack of available antibodies for flow cytometry and Western blot techniques. Our group has generated a full set of fluorescent ligands against the A1 (33, 34), A_2A_ (35, 36), A_2B_ (37) and A_3_ (38) adenosine receptors based on small-molecule antagonist drugs. Using these we have demonstrated A_2A_ (36) and A_2B_ (37) expression in our macrophage models. Crucially, most studies have looked at A_2A_ or A_2B_ receptors in isolation, using either knockout models or single-receptor pharmacological inhibition, with limited study of the relative contributions of A_2A_ and A_2B_ when co-expressed. Whilst it is established that both the A_2A_ and A_2B_ receptors modulate myeloid cell function (39), the respective level of compensatory function vs non-redundant function has not been explored nor has it been proven that blockade of only A_2A_ receptors will not mitigate the effect of adenosine on myeloid cells in the TME.

We hypothesised that adenosine polarises human macrophages towards an immunosuppressive phenotype by signalling through both the A_2A_ and A_2B_ receptors, and that dual receptor blockade is required to reverse this. Here we present evidence that dual A_2A_ and A_2B_ receptor antagonism is required to reverse the effects of NECA on macrophages and prevent deleterious downstream effects on cells important for anti-tumour immunity.

## 2 Methods

### 2.1 Reagents

Endotoxin free reagents were used throughout. All media were based on RPMI 1640 with L-glutamine (Sigma). Macrophage medium contained v/v 10% FCS and 1% sodium pyruvate (Sigma), T cell medium v/v 10% FCS, 1% HEPES, 1% sodium pyruvate, 1% non-essential amino acids and 20µM 2-mercaptoethanol (all Sigma). Cytokines were recombinant human (rh) Granulocyte-macrophage colony-stimulating factor (GM-CSF, 20U/ml, Peprotech), rh Macrophage colony-stimulating factor (M-CSF, 10ng/ml, Immunotools), lipopolysaccharide (LPS, 1mg/ml, Sigma), interferon gamma (IFNγ, 1000U/ml, R&D). NECA (5′-(N-Ethylcarboxamido)- adenosine), SCH442416 (A_2A_ inhibitor) and PSB603 (A_2B_ inhibitor) were all from Tocris. Phytohaemaglutinin (PHA, 4μg/ml, Sigma).

### 2.2 Ethics approval and consent to participate

All donors were recruited in accordance with Good Clinical Practice and the Declaration of Helsinki and provided written informed consent. Ethical approval was provided by University of Nottingham Medical School Ethics Committee (ref 161-1711).

### 2.3 Macrophage generation

PBMC were separated from heparinised blood immediately after venepuncture by density centrifugation over Histopaque 1077 (Sigma). CD14+ cells were isolated using Miltenyi CD14+ microbeads (purity >95% by flow cytometry, **Supplementary Figure 1**). CD14+ monocytes were resuspended at 1x10^6^/ml and plated in ultra-low attachment plates (Corning costar) at either 5x10^6^/well (6 well plate) or 1x10^6^/well (24 well plate). For ‘M1’-type macrophages, GM-CSF was added at day 0 at 20U/ml and for ‘M2’-type macrophages, M-CSF was added at day 0 at 10ng/ml. Macrophages were fed at day 4 with equal volume of cytokine-supplemented medium. In some experiments, NECA, SCH442416 and/or PSB603 (all 1μM) were added to macrophage medium at d0 and d4 of differentiation. On day 7, macrophages were harvested by incubation on ice for 15 minutes in cold endotoxin-free PBS (Sigma), resuspended in macrophage medium (or T cell medium for co-culture experiments), plated and rested for 2 hours at 37°/5%CO_2_ prior to any drugs/stimulation. Macrophages for flow cytometry prior to stimulation were used immediately without re-plating.

### 2.4 *In vitro* stimulation

Macrophages were resuspended at 5x10^5^/ml and plated at 5x10^4^ per well in a standard 96-well plate. After resting for 2 hours macrophages were stimulated with LPS (1mg/ml) + IFNγ (1000U/ml) (GM-CSF/’M1-like’ macrophages) or LPS alone (M-CSF/’M2-like’ macrophages). In some experiments, NECA (1nM-10μM) and/or adenosine receptor inhibitor drugs (1μM) were added for 1 hour prior to stimulation. After 24hrs, supernatants were harvested for ELISA for IL-12p70 (BD Biosciences) and IL-10 (R&D) or for multiplex analysis using a custom-designed Luminex Human Magnetic Assay (R&D Systems). For flow cytometric analysis of stimulated macrophages, cells were harvested at 48hrs by scraping.

### 2.5 PBL:macrophage co-culture

Allogeneic peripheral blood leucocytes (PBL, CD14- fraction from monocyte isolations, frozen immediately after isolation at 2x10^7^/ml in 90% FCS + 10% DMSO) were defrosted and rested overnight at 2x10^6^/ml in T cell medium in 6 well plates (Costar). The next day, day 7 macrophages were harvested, plated at 1x10^3^, 3.3x10^3^, 1x10^4^ or 2x10^4^/well in 100μl T cell medium in a 96-well plate and rested for 2 hours at 37°/5%CO_2._ PBL were then harvested, counted by haemocytometer, resuspended and added at 1x10^5^ per well. PHA was added at 4μg/ml in a final volume of 200μl. NECA was not included in the co-culture in order to assess the impact of the NECA-conditioned macrophages alone. On day 4, 50μl supernatant was removed for ELISA for IFNγ (BD Biosciences), IL-10 and IL-17 (both R&D). 50μl T cell medium containing ^3^H-Thymidine was added immediately and cells cultured for a further 18hr then frozen at -20 °C. Cells were subsequently transferred to 96-well unifilter microplates (PerkinElmer), dried overnight at room temperature then Microscint-O (PerkinElmer) added and read on a TopCount NXT (PerkinElmer). PBL without PHA stimulation were used as negative control and had undetectable levels of all cytokines and negligible thymidine incorporation (not shown).

### 2.6 Flow cytometry

Antibodies (Miltenyi Biotec) were used at 1:50 dilution with fluorescence-minus-one (with matched isotype) or isotype control. For multicolour experiments, a compensation matrix was generated using single-stained anti-REA compensation beads (Miltenyi Biotec 130-104-693). Cells were washed (300g, 10mins) in cold FACS buffer (1% FBS, 2mM EDTA, and 0.01% sodium azide in PBS), resuspended and stained for 15minutes at 4 °C in the dark before washing, resuspension in 200μl cold FACS buffer and acquisition on a MACSQuant 10 (Miltenyi Biotec). Data were analysed using FlowJo v10. Antibodies (Miltenyi Biotec unless otherwise stated) were CD14 FITC (130-110-518), CD14 PerCP/Cy5.5 (Biolegend, 367110), CD68 PE-Vio770 (130-114-462), HLADR VioGreen (130-111-795), CD163 FITC (130-112-132), mouse IgG1 PerCP/Cy5.5 (Biolegend 400150), REA controls human IgG1 VioGreen (130-113-456), IgG1 FITC (130-113-449) and IgG1 PE-Vio770 (130-113-440). Gating strategy for flow cytometry is shown in **Supplementary Figures 1**, **2**.

### 2.7 Statistics

Data were analysed using Graphpad Prism with tests as indicated in figure legends. Summary data for multiple donors was normalised where appropriate to control condition for each donor then mean +/- SEM calculated for all donors together. In all figures, *=p<0.05, **=p<0.005, ***=p<0.001.

## 3 Results

### 3.1 Adenosine analogue NECA modulates the function of both fully differentiated and differentiating human macrophages

The effect of the adenosine analogue NECA on fully differentiated and differentiating human macrophages was initially determined. NECA was employed throughout this study due to its extended half-life compared to native adenosine. Both GM-CSF-differentiated macrophages (GMCSF-Mφ) and MCSF-differentiated macrophages (MCSF-Mφ) upregulated CD68 and downregulated CD14 relative to freshly isolated CD14+ monocytes (**Supplementary Figure 1**; **Figure 1A**). Following stimulation, GMCSF-Mφ displayed an HLA-DRhiCD163lo phenotype, whereas MCSF-Mφ displayed an HLA-DRloCD163hi phenotype (**Figure 1B**). GMCSF-Mφ produced abundant IL-12 and smaller amounts of IL-10, whilst MCSF-Mφ produced little/no IL-12 and higher levels of IL-10 (**Figure 1C**).

**Figure 1:**
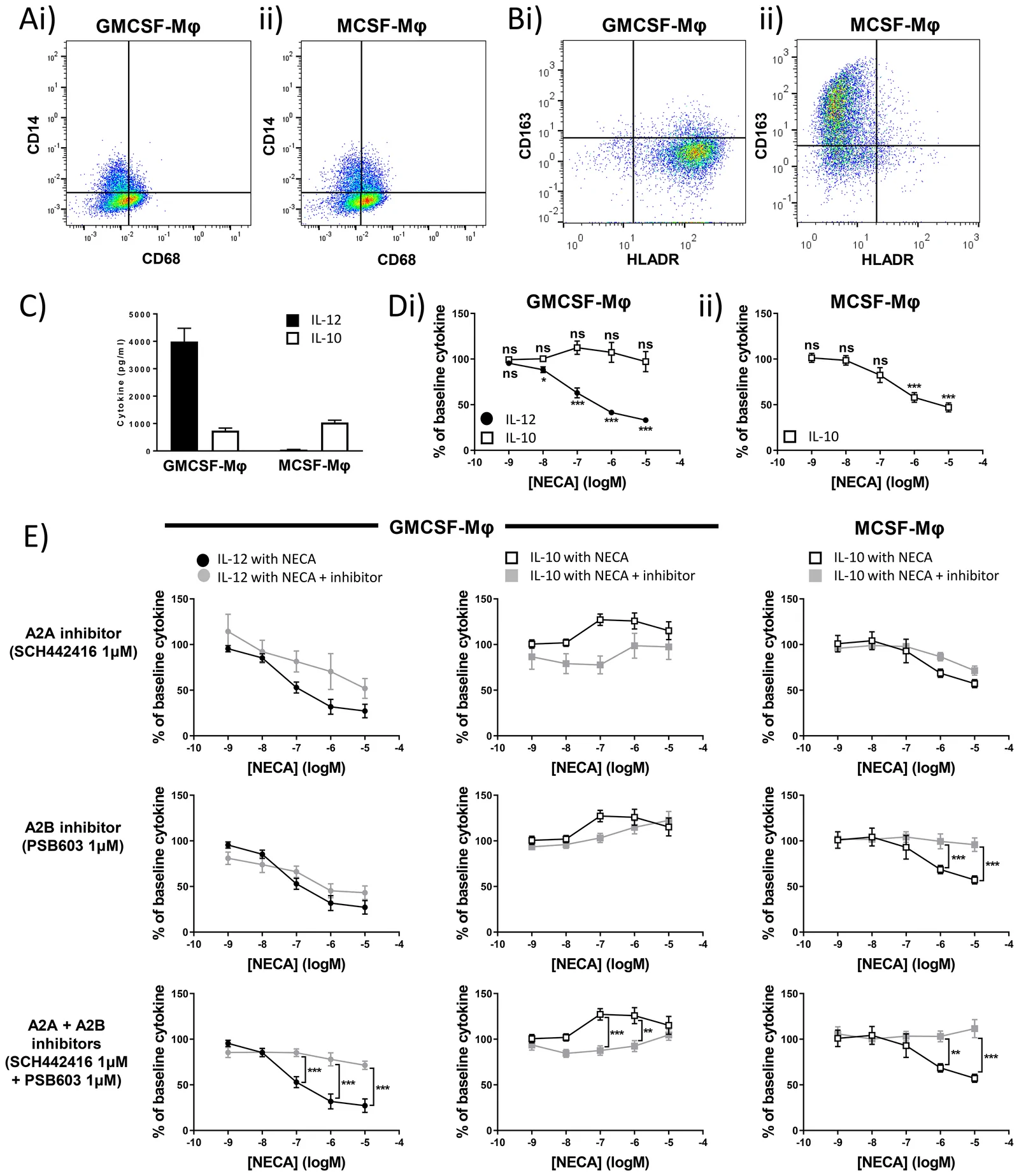
NECA modulates cytokine production by fully differentiated human macrophages via both A_2A_ and A_2B_ receptors. **(A)** Macrophages differentiated in GM-CSF (GMCSF-Mφ, i) or M-CSF-containing medium (MCSF-Mφ, ii) were harvested, stained for CD14 and CD68 expression and analysed by FACS (representative donor of n=3). **(B)** GMCSF-Mφ **(i)** and MCSF-Mφ **(ii)** were were stimulated with LPS or LPS/IFNγ respectively, harvested, stained for CD163 and HLADR expression and analysed by FACS after 48hrs (representative donor of n=3). **(C)** GMCSF-Mφ and MCSF-Mφ were stimulated as in **(B)** and secretion of IL-12 (black bar) and IL-10 (white bar) measured by ELISA in culture supernatants after 24hrs. (representative donor of n=25, mean +/- SEM of triplicate stimulation). **(D)** GMCSF-Mφ **(i)** and MCSF-Mφ **(ii)** were treated with increasing concentrations of NECA prior to stimulation and cytokine analysis as in **(C)** (summary of 9 donors each normalised to no NECA, mean +/- SEM, one-way ANOVA with *post-hoc* Dunnett’s multiple comparison compared to vehicle controls in the absence of NECA). **(E)** GMCSF-Mφ and MCSF-Mφ were treated with an A_2A_ receptor antagonist (SCH442416, 1μM), an A_2B_ receptor antagonist (PSB603, 1μM) or both prior to addition of NECA, stimulation and cytokine analysis as in **(A)** (summary of 5 donors each normalised to no NECA, mean +/- SEM, mixed-effects analysis with Sidak’s multiple comparisons test). *p<0.05, **p<0.005, ***p<0.001.

Exposure of differentiated MCSF-Mφ to NECA one hour prior to activation reproducibly produced a significant concentration-dependent decrease in IL-10 (**Figure 1Dii**, p<0.0001 by one-way ANOVA). This finding is somewhat unexpected and contrasts with murine studies (26). NECA treatment of differentiated GMCSF-Mφ significantly reduced IL-12 production whilst leaving IL-10 unaffected (**Figure 1Di**, p<0.0001 and p=0.66 by one-way ANOVA respectively).

The A_2A_ is the most important adenosine receptor on T cells (16) and is currently the main clinical adenosine receptor target. In contrast, both A_2A_ and A_2B_ receptors are expressed in myeloid cells (30–32). We therefore used selective small molecule inhibitors of the A_2A_ and A_2B_ receptors (SCH442416 and PSB603 respectively) to determine their relative contribution to the effects of NECA on human macrophages. For the first time we demonstrated that simultaneous blockade of both A_2A_ and A_2B_ receptors was required to recover IL-12 secretion in GMCSF-Mφ (**Figure 1E** left panel, bottom, p<0.001). Crucially, given that current clinical strategies predominantly target A_2A_ alone, blocking either receptor individually did not restore IL-12 production in GMCSF-Mφ (**Figure 1E** left panel, top and middle). Dual A_2A_/A_2B_ blockade also significantly reduced IL-10 secretion in GMCSF-Mφ (**Figure 1E** middle panel, bottom). In MCSF-Mφ, the effect of NECA on IL-10 production was largely reversed by blockade of A_2B_ alone (**Figure 1E** right panel, middle, p<0.001 at NECA concentrations ≥1μM), whilst A_2A_ blockade alone had very little effect (**Figure 1E** right panel, top). Treatment of macrophages with A_2A_ or A_2B_ antagonist alone prior to activation did not modulate cytokine production (not shown). Thus, the ‘M2’ polarising effect of NECA on differentiated GMCSF-Mφ was only completely reversed by blockade of both A_2A_ and A_2B_ receptors, but blockade of A_2B_ alone largely reversed the effect of NECA on differentiated MCSF-Mφ.

Upon exiting the vasculature and entering the TME, monocytes would encounter markedly higher levels of adenosine throughout their differentiation to TAM. Therefore, we studied the effect of NECA on the differentiation of macrophages in order to better reflect conditions experienced in the TME. As with post-differentiation exposure, NECA present during differentiation significantly suppressed IL-12 production by GMCSF-Mφ (**Figure 2A**). Unlike post-differentiation exposure, however, NECA present during differentiation substantially increased IL-10-secreting capacity in both GMCSF-Mφ and MCSF-Mφ, consistent with previous murine data (26) (**Figure 2A**). This resulted in a significant reduction in the IL-12:IL-10 ratio in NECA-conditioned GMCSF-Mφ (**Figure 2Bi**, p<0.05) and a mean 2.10-fold increase in IL-10 production (p<0.05, **Figure 2Bii**).

**Figure 2:**
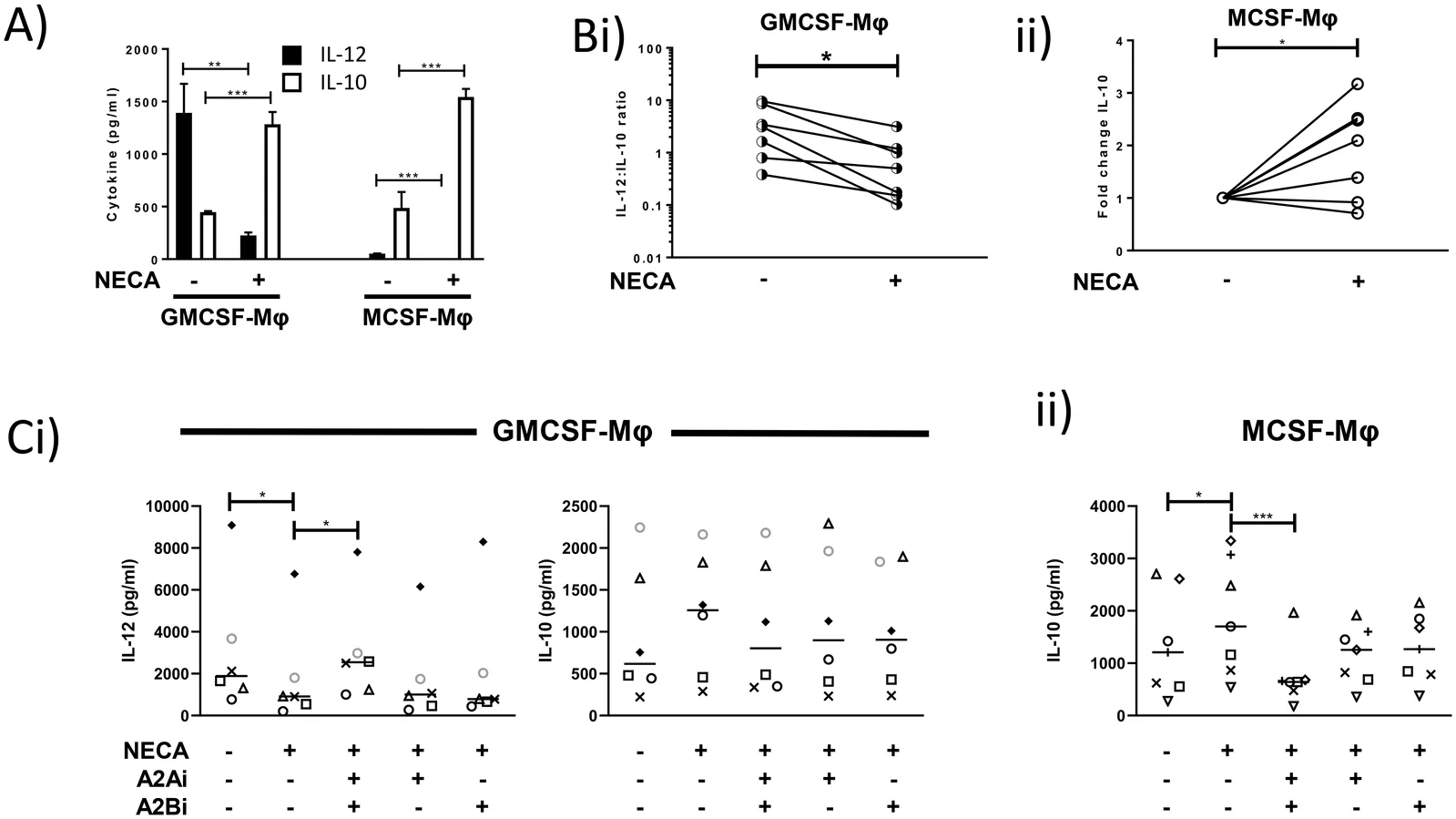
NECA-conditioning of differentiating human macrophages polarises towards ‘M2’ type via both A_2A_ and A_2B_ receptors. **(A)** Macrophages differentiated in GM-CSF (GMCSF-Mφ) or M-CSF-containing medium (MCSF-Mφ) +/- NECA (1μM) were stimulated with LPS or LPS/IFNγ respectively and secretion of IL-12 and IL-10 measured by ELISA in culture supernatants after 24hrs (representative donor of n=13, mean +/- SEM of triplicate stimulation, 2-tailed paired students T test). **(B)** i) IL-12:IL-10 ratio for GMCSF-Mφ and ii) fold-change IL-10 for MCSF-Mφ both differentiated with/without NECA, stimulated and analysed as in **(A)** (both n=7, 2-tailed paired students T test). **(C)** Macrophages differentiated, stimulated and analysed as in **(A)** +/- A_2A_ inhibitor (A_2A_i, SCH442416 1μM), A_2B_ inhibitor (A_2B_i, PSB603 1μM) or both during differentiation. Summary of 6 donors (GMCSF-Mφ) and 7 donors (MCSF-Mφ) normalised to no-NECA control (mean +/- SEM, Friedman Test with Dunn’s post test). Each symbol represents an individual donor. *p<0.05, **p<0.005, ***p<0.001.

### 3.2 Simultaneous blockade of A_2A_ and A_2B_ receptors is required to reverse the effect of NECA on differentiating human macrophages

Having established that both the A_2A_ and A_2B_ receptors mediate the effects of adenosine on fully differentiated macrophages, we investigated how signalling through these receptors modified macrophage differentiation from monocytes. In agreement with the contribution of adenosine receptors in fully differentiated macrophages, combined A_2A_ and A_2B_ blockade was required to recover IL-12 production by NECA-conditioned GMCSF-Mφ (**Figure 2Ci**, p<0.005). Notably, single A_2A_ or A_2B_ blockade did not recover IL-12 production in GMCSF-Mφ (**Figure 2Ci**).

In MCSF-Mφ, where NECA-conditioning significantly increased IL-10 production, combined A_2A_ and A_2B_ blockade significantly reduced IL-10 production (**Figure 2Cii**), whilst single A_2A_ or A_2B_ blockade had no significant effect. In both GMCSF-Mφ and MCSF-Mφ, there was no significant effect of adenosine receptor blockade alone on macrophage differentiation (not shown).

### 3.3 NECA has widespread effects on human macrophage function, mediated via both A_2A_ and A_2B_ receptors.

Having established the impact of adenosine receptor inhibition on macrophage polarisation we investigated the broader impact on macrophage secretory phenotype. The presence of NECA during MCSF-Mφ differentiation markedly reduced the secretion of IL-23, CCL-22, IL-6, TNFα, FGF-b, IL-1β and MMP2 (**Figure 3**). For the majority, significant recovery was again only seen with dual A_2A_/A_2B_ blockade. VEGF-A was markedly induced by NECA-differentiation in MCSF-Mφ and again only fully reversed by dual A_2A_/A_2B_ blockade. There was no significant effect on VEGF-C or VEGF-D (not shown). MMP2 production by NECA-differentiated MCSF-Mφ was also significantly increased. Notably, GMCSF-Mφ were less impacted by NECA-polarisation than MCSF-Mφ, with significant alterations only seen in Eotaxin 2 and CCL17 (**Supplementary Figure 1**). Baseline comparison between control GMCSF-Mφ and MCSF-Mφ showed that, as expected, GMCSF-Mφ produce more pro-inflammatory cytokines (IL-23, IL1β, IL-6 and TNFα) whilst MCSF-Mφ produce more MMP7, MMP9 and VEGF-A (**Supplementary Figure 4**).

**Figure 3:**
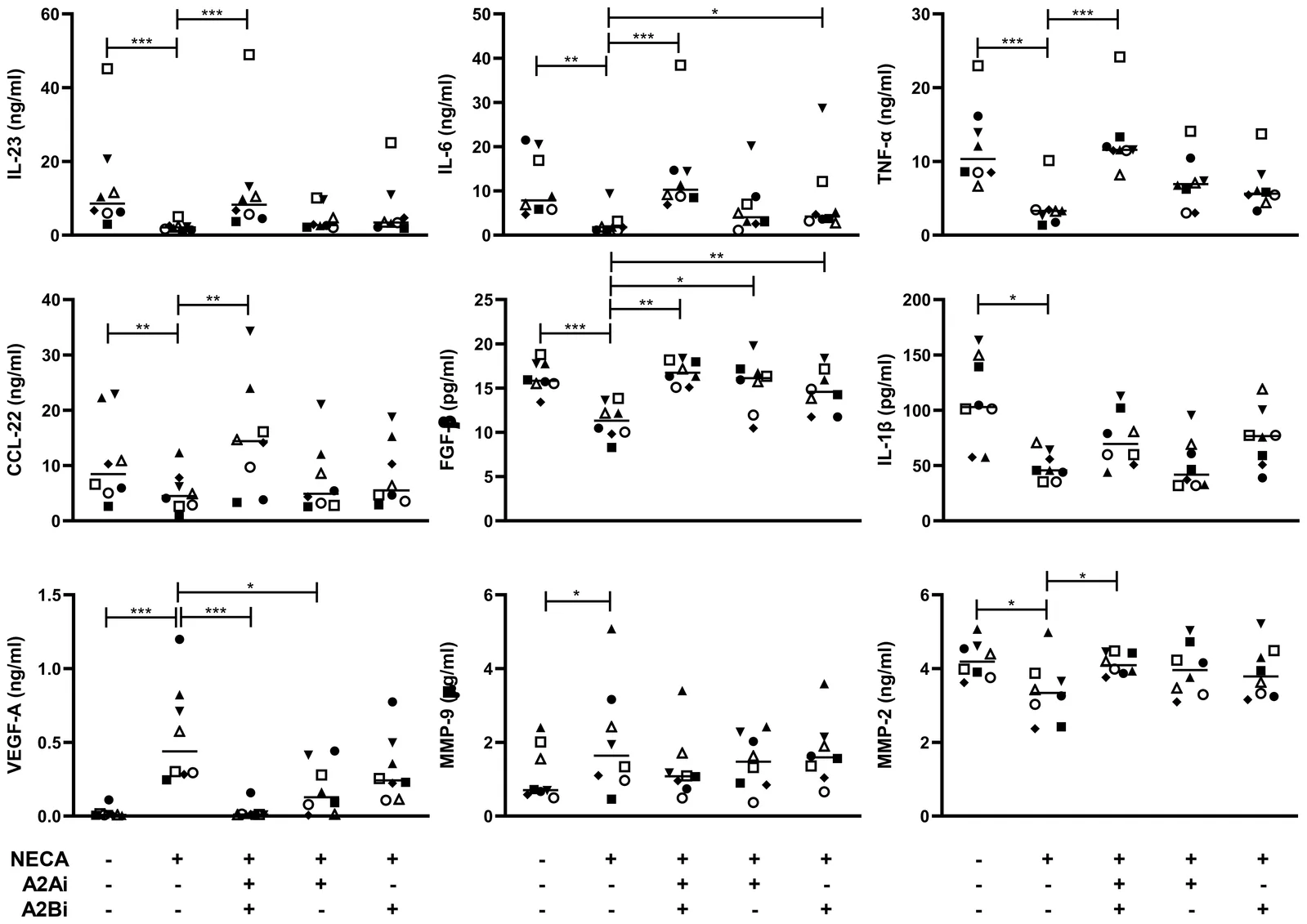
NECA-conditioning of human M-CSF macrophages alters multiple cytokines and chemokines. M-CSF macrophages conditioned with NECA (1μM) +/- A_2A_ inhibitor (A_2A_i, SCH442416 1μM) and/or A_2B_ inhibitor (A_2B_i, PSB603 1μM) were stimulated with LPS for 24hrs and supernatants collected for Luminex analysis. Shown are single determinations of secretion for 8 independent donors (each symbol=one donor) with line at median. Statistical analysis using Friedman test with Dunn’s post –test, *p<0.05, **p<0.005, ***p<0.001.

### 3.4 NECA-induced repolarisation of human macrophages impacts on downstream T cell function

To determine whether NECA-polarisation of macrophages exerted a downstream impact on T cells, we carried out macrophage-PBL co-cultures. NECA-conditioned MCSF-Mφ significantly and dramatically suppressed the ability of T cells to secrete IFNγ compared to control MCSF-Mφ (**Figures 4Ai, Biii**, p< 0.05). Importantly, the suppression of Th1 responses was completely reversed by A_2A_/A_2B_ antagonism during macrophage differentiation (**Figure 4AI, Aiii**, p=0.0410; **Supplementary Figure 5**), confirming the importance of dual A_2A_/A_2B_ blockade. The suppression of IFNγ was seen more markedly as the proportion of NECA-conditioned MCSF-Mφ increased (**Figure 4B**; **Supplementary Figure 3**). In contrast, IL-10 and IL-17 were unaffected by NECA-conditioning in MCSF-Mφ (**Figures 4Ai, iii**). In keeping with the more modest effects seen on other cytokines and chemokines, NECA-conditioning of GMCSF-Mφ did not affect Th1 responses (**Figures 4Ai, iii**). Stimulation of A_2A_ receptors in T cells suppresses their ability to proliferate (40) so a potential mechanism for the reduction in IFNγ was a reduced absolute number of lymphocytes due to carry-over of NECA. However, we demonstrated that NECA-conditioned macrophages did not alter subsequent T cells proliferation (**Figure 4Ci, ii**), confirming that the reduced IFNγ production was due to alterations in lymphocyte phenotype in response to NECA-differentiated macrophages.

**Figure 4:**
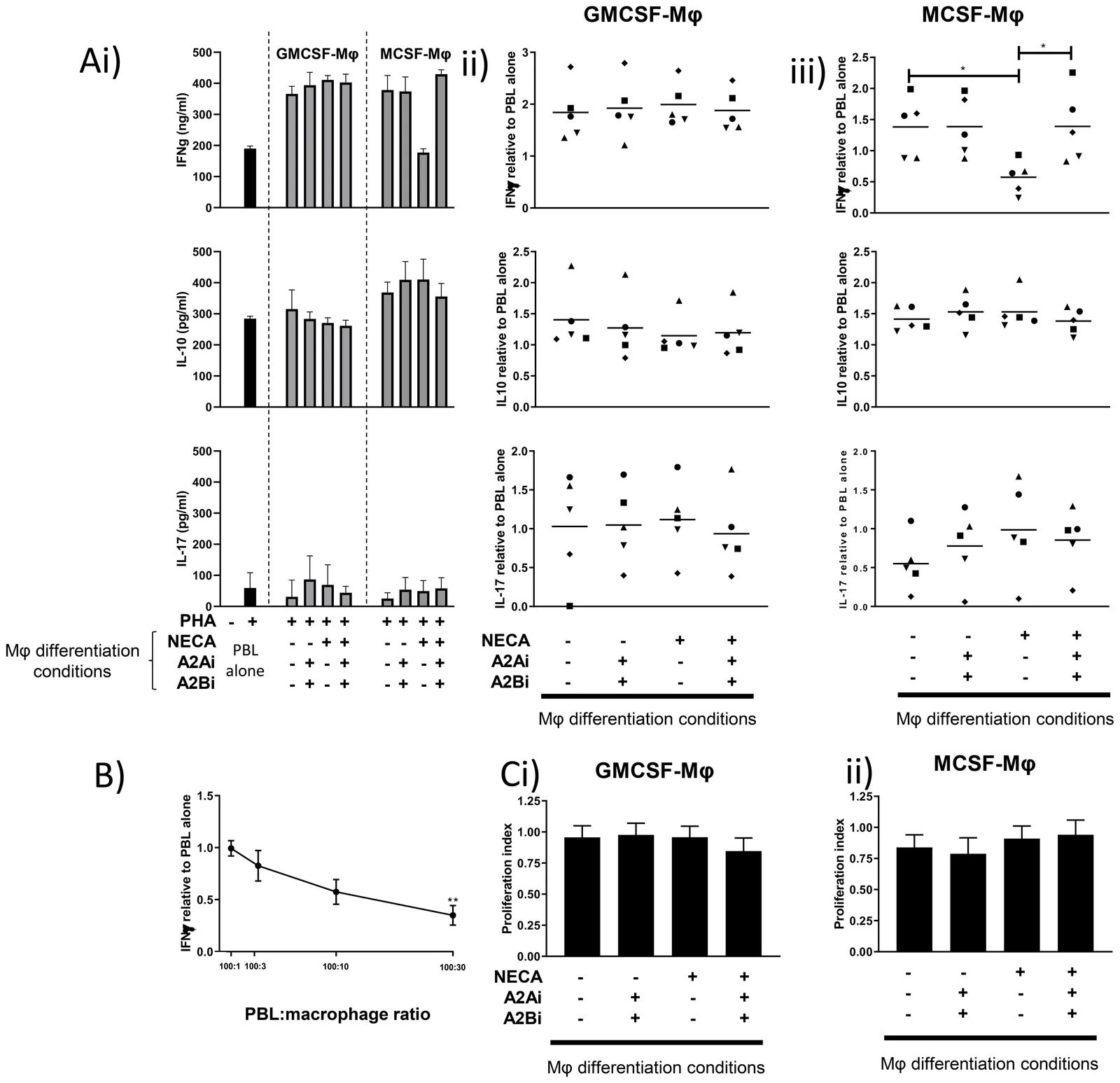
NECA acts via A_2A_ and A_2B_ receptors during differentiation to polarise human macrophages to impair T cell IFNγ responses. Macrophages were differentiated in NECA-containing medium (1μM) +/- A_2A_ inhibitor (A_2A_i, SCH442416 1μM) and A_2B_ inhibitor (A_2B_i, PSB603 1μM) with either GM-CSF (GMCSF-Mφ) or M-CSF (MCSF-Mφ), harvested then co-cultured for 5 days with peripheral blood lymphocytes (PBL) along with PHA stimulation at 100:20, 100:10, 100:3 and 100:1 PBL:macrophage ratios. At 4 days, 50μl supernatant was collected for cytokine analysis via ELISA for IFNγ, IL-10 and IL-17, and replaced with 50μl medium containing thymidine for final 18hrs for proliferation analysis. **(A)** IFNγ (top), IL-10 (middle) and IL-17 (bottom) secretion of 100:10 PBL:macrophage ratio i) mean +/- SEM of triplicate co-culture from 1 representative donor of 5 for GMCSF-Mφ and MCSF-Mφ, ii) & iii) summary of each donor normalised to PBL + PHA alone GM-CSF and MCSF respectively, shown is mean for each donor (each symbol=one donor, n=5) with line at mean (One-way ANOVA with Dunnett’s multiple comparison test). **(B)** IFNγ production by PBL co-cultured with NECA-conditioned MCSF-Mφ at different PBL:macrophage ratios (mean +/- SEM of data normalised as in Aiii) (n=5 donors) (One-way ANOVA with Dunnett’s multiple comparison test). **(C)** Proliferation analysis normalised to PBL + PHA alone for each donor (mean +/- SEM) for GMCSF-Mφ (i) and MCSF-Mφ (ii) (n=5 donors) at 100:10 PBL:macrophage ratio. *p<0.05, **p<0.005, ***p<0.001.

## 4 Discussion

Our data show for the first time that dual A_2A_ and A_2B_ receptor antagonism is required to completely reverse the effects of NECA on human macrophages *in vitro*. Furthermore, we demonstrate that the cytokine and chemokine alterations induced by NECA during macrophage differentiation have significant critical downstream effects on the adaptive immune cells which are required for tumour immunotherapy to be effective. The fact that these cytokine and chemokine changes were only reversed by dual A_2A_ and A_2B_ receptor blockade suggests that in tumours with significant macrophage infiltration, A_2A_ blockade alone will not be sufficient to repolarise those macrophages to support tumour immunotherapy. Prior work in murine macrophages using A_2A_-knockout models showed that adenosine can suppress TNFα via A_2B_ alone (30). However, in wild-type mice with expression of both A_2A_ and A_2B_ receptors, an A_2B_ receptor antagonist alone could not block the effects of adenosine, in keeping with our data. Another group also demonstrated in murine macrophage models that both A_2A_ and A_2B_ could mediate effects of adenosine when the other was not present (41). In human lung macrophages, NECA reduced TNFα production, and A_2A_ antagonism only partially reversed this effect (31). A separate study of human monocytes likewise found only partial recovery of TNFα production with A_2A_ antagonism alone (28). However, crucially, none of these studies nor any others have examined the relative importance of A_2A_ and A_2B_ receptors when physiologically expressed and acting together in either murine or human macrophages. Our work is therefore the first to conclusively show that the effect of adenosine is mediated via both A_2A_ and A_2B_ receptors in human macrophages *in vitro*.

When A_2A_ and A_2B_ receptors are co-expressed, the lower-affinity A_2B_ receptor tends to dictate the response to NECA and adenosine (42, 43). For example, in HEK 293G cells, the A_2A_ receptor contribution to cAMP responses to adenosine and NECA is very small, and the effect of A_2A_ receptor antagonism is limited (42). Small signals can, however, be observed with selective A_2A_ agonists (42). We have generated both A_2A_- and A_2B_-specific fluorescent ligands (35) and are using them to characterise receptor levels in human macrophages generated under different conditions, including hypoxia and adenosine exposure. Differing ratios of A_2A_ to A_2B_ expression in GMCSF-Mφ versus MCSF-Mφ may explain the differential response to single-antagonist treatment we observed. Notably, A_2B_ expression exceeds A_2A_ in resting human lung macrophages (31). Most clinical trials in cancer targeting adenosine receptors have combined A_2A_ blockade with checkpoint inhibition. Further preclinical work is now needed to test whether dual A_2A_/A_2B_ blockade offers additional benefit over A_2A_ blockade alone when combined with checkpoint inhibition. There is a dual A_2A_/A_2B_ antagonist, AB928 for which phase 1b/2 studies are ongoing and showing encouraging efficacy (44). Another compound targeting the A_2B_ receptor alone is currently in trials for non-small cell lung cancer after demonstration that A_2B_ blockade in combination with anti-PD-1 was more beneficial than anti-PD-1 alone (45). A murine paper shows that combination of caffeine (a non-specific adenosine receptor antagonist) with anti-PD-1 therapy leads to better tumour control in a carcinogen-induced tumour model and the B16F10 model than either therapy alone, with accompanying reduction in Treg infiltration and increase in both CD4 and CD8 cells in the tumour (46). Another paper, whilst showing that caffeine was beneficial when combined with T cell therapy, showed more substantial benefits with specific pharmacological inhibition of A_2A_ alone (47). This suggests that whilst dual A_2A_/A_2B_ blockade has promise, specific and high affinity pharmacological targeting will be required to realise the full potential of this approach. The latter paper was predominantly exploring A_2A_ receptor knockout/blockade in T cells but does also contain data supporting the concept that dual knockout of A_2A_/A_2B_ even in T cells has an additional benefit over A_2A_ blockade alone (47). In addition to the role of A_2B_ receptors on immune cells, there is renewed interest in this receptor more broadly within tumours as a potential therapeutic target (48) as well as appreciation that both the A_2A_ and A_2B_ receptors may need to be targeted (49). Furthermore, in a study investigating T cell-adenosine interactions, whilst T cell A_2A_ receptor expression was generally higher than A_2B_, it is notable that in T cell populations that expressed higher than normal A_2B_ levels, A_2B_ inhibition could reverse adenosine-mediated suppression of effector function equally effectively as A_2A_ inhibition (17). Overall, our data and these other studies support the concept that dual A_2A_/A_2B_ blockade may be required for cancer immunotherapy rather than targeting the A_2A_ receptor alone.

Our multiplex analysis of secreted cytokines and chemokines shows that NECA has a much more marked effect on MCSF-Mφ than GMCSF-Mφ. GM-CSF is a strongly pro-inflammatory cytokine, and appears to ‘protect’ the macrophages from polarisation towards an M2 phenotype. Given that MCSF-Mφ are more representative of TAMs, the very marked effect of NECA seen on multiple secreted factors in this type of macrophage confirms the importance of developing methods to block the action of adenosine on macrophages in the TME. Whilst several of the cytokines and chemokines altered by NECA were T cell polarising cytokines (IL-10, TNFα, IL-6 and IL-23), one of the most marked changes was in VEGF-A. It is unsurprising that NECA induces macrophages to produce more VEGF-A, since adenosine is upregulated in hypoxic environments and stimulation of angiogenesis is a mechanism to reverse hypoxia caused by inadequate blood supply. Previous work also noted an increase in VEGF production by human macrophages treated with adenosine (29). They studied the impact of single adenosine receptor blockade and saw a significant reduction with A_2A_ blockade alone, however the combination of A_2A_ and A_2B_ blockade was not examined. Another study showed that in a murine model of oxygen-induced retinopathy, macrophage-specific AdorA_2A_ deletion did not reduce pathological neovascularization (50). One interpretation is that macrophages are not involved in this pathogenesis, but another is that adenosine also acts via A_2B_ in those macrophages and therefore deletion of only A_2A_ would not block its effects. In support of this hypothesis, there is evidence that the A_2B_ receptor is the predominant adenosine receptor controlling VEGF production (51). CCL22, which was significantly reduced by NECA-conditioning, induces chemotaxis of T cells by binding to the CCR4 receptor. Decreased levels of CCL22 produced by TAMs exposed to high levels of adenosine would therefore impair migration of T cells into the tumour, which would be detrimental to current tumour immunotherapeutic effects. This multi-parameter effect of NECA on human macrophages underscores the importance of targeting adenosine receptors on TAMs to support immunotherapy.

We saw a differential effect of NECA-conditioning during differentiation versus NECA treatment after differentiation of macrophages, suggesting that receptor expression and/or function varies in different types of myeloid cells. Our finding that IL-10 decreased with NECA-treatment in fully differentiated MCSF-Mφ was unexpected. Another paper has also noted a decrease in IL-10 when monocytes were treated with the adenosine analogue NECA immediately prior to stimulation (52). As well as differential effects in different types of myeloid cells, it is likely that other cell types or culture conditions can modulate the effects of adenosine on human macrophages. For this reason, we are working to extend these findings into multi-cellular co-culture systems and explore the effects of other factors which are commonly encountered within the TME or during anti-cancer treatment, including hypoxia, extracellular matrix and radiotherapy. Hypoxia is particularly relevant in this context, given published evidence that A_2B_ receptors are preferentially upregulated in myeloid cells exposed to hypoxia (32).

In summary, this work is the first to clearly demonstrate that the A_2A_ and A_2B_ receptors are both non-redundant in mediating the effect of the adenosine analogue NECA on differentiating human macrophages *in vitro*, altering their functional phenotype in a way that modulates downstream immune responses. Dual targeting of A_2A_ and A_2B_ should therefore be explored further *in vivo* and in clinical trials as a therapeutic strategy for cancer immunotherapy.

## Data Availability

The raw data supporting the conclusions of this article will be made available by the authors, without undue reservation.
