# Supplementary material for: Dual blockade of adenosine A2A and A2B receptors is required to reverse NECA-induced immunosuppression in human macrophages: Implications for cancer immunotherapy
Source: Front Immunol. 2026 Sep 14;17:1845291. doi: 10.3389/fimmu.2026.1845291 (PMC13616649; doi:10.3389/fimmu.2026.1845291)
Supplement: Supplementary file 1 [file DataSheet1.pdf]

Supplementary Figure 1

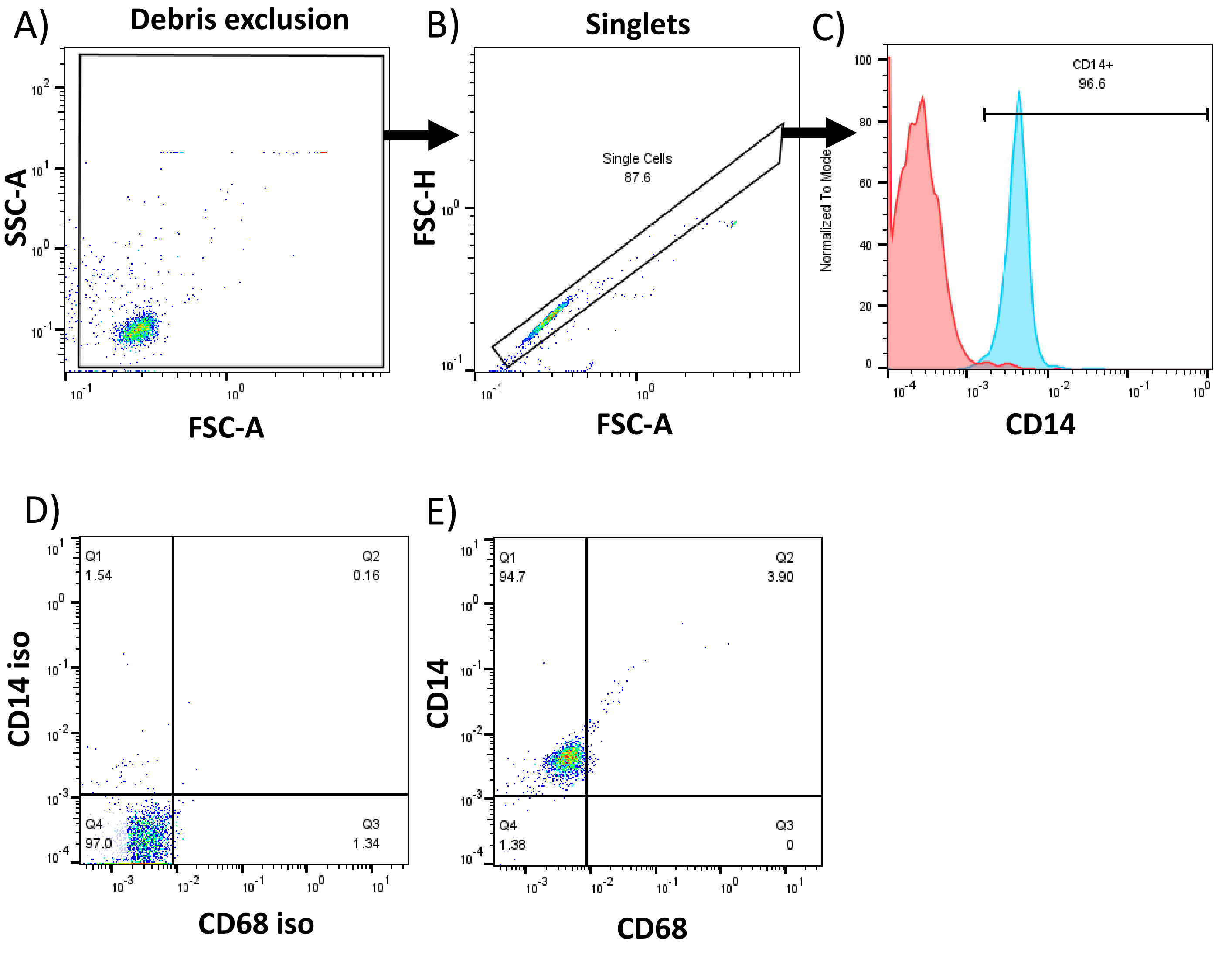

Monocyte purity (CD14+) and CD68 expression:

A) Debris exclusion using FSC-A vs SSC-A
B) Doublet exclusion using FSC-A vs FSC-H
C) CD14 purity- isotype gate set at ~1% then overlaid on CD14 stained monocytes
D) Gating on isotype for CD14 and CD68 assessment from singlets (B)
E) CD14 and CD68 expression from singlets (B)

Supplementary Figure 2A

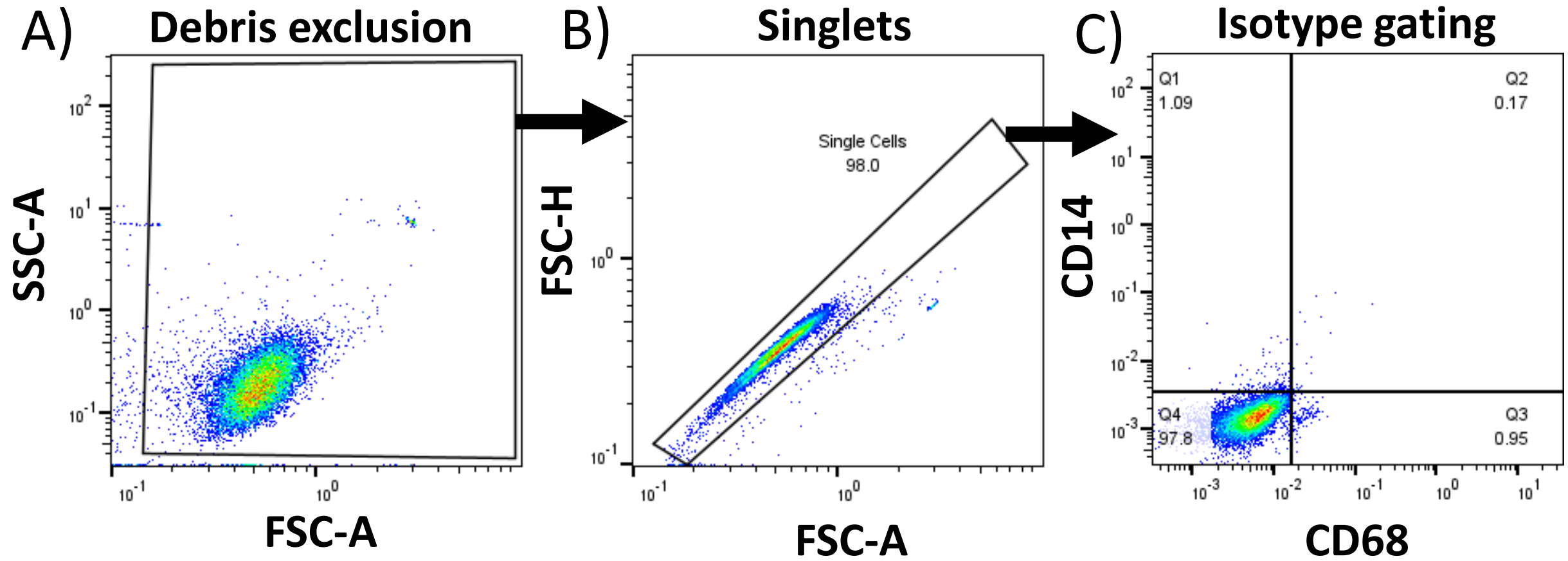

Gating strategy for unstimulated macrophage phenotyping.

A) Debris exclusion using FSC-A vs SSC-A

B) Doublet exclusion using FSC-A vs FSC-H

C) Gating for CD14 and CD68 positivity on singlet population using dual isotype controls

Supplementary Figure 2B

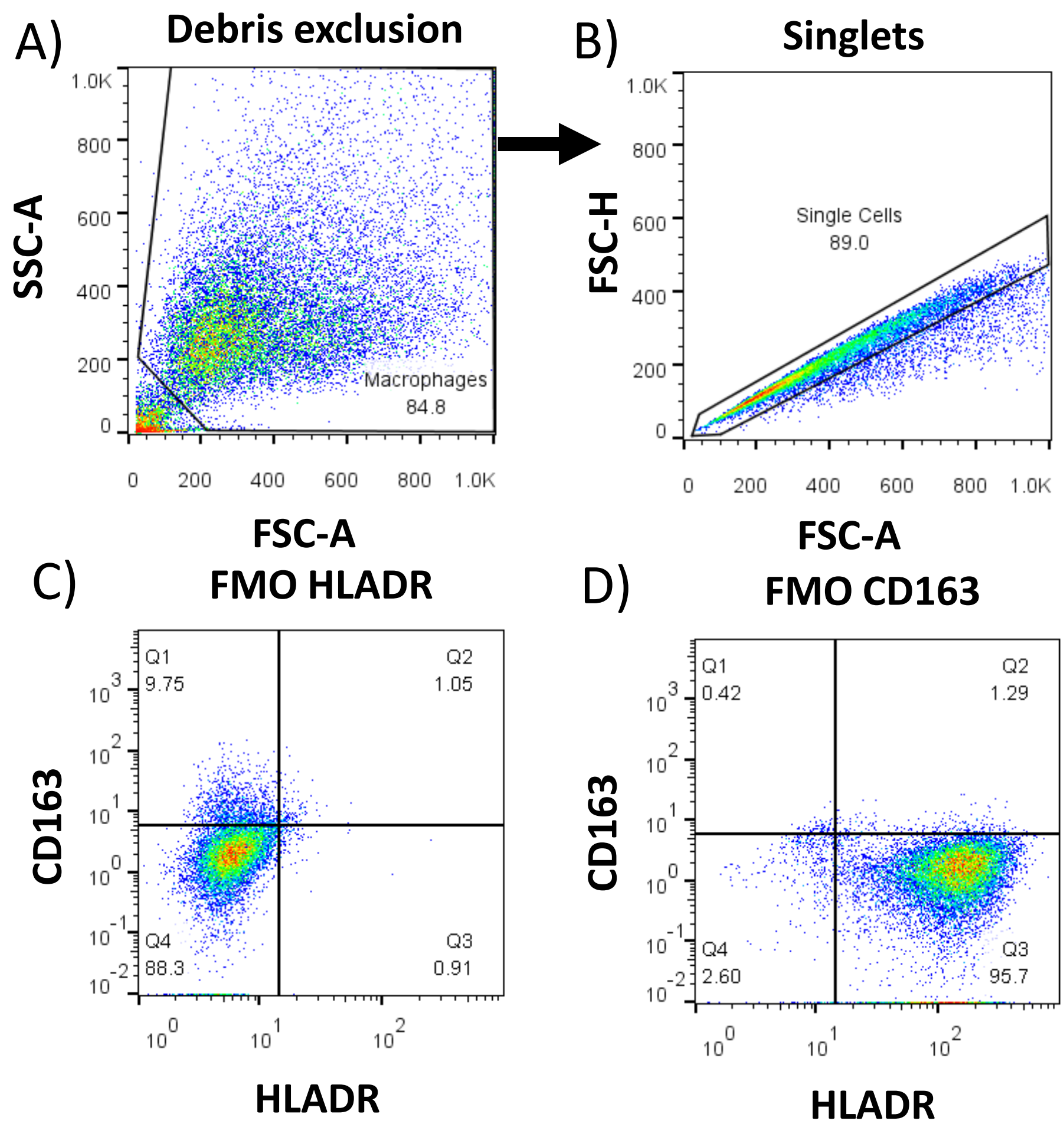

Gating strategy for stimulated macrophage phenotyping.

A) Debris exclusion using FSC-A vs SSC-A

B) Doublet exclusion using FSC-A vs FSC-H

C) Gating for HLADR positivity on singlet population using fluorescence-minus-one (FMO) sample for HLADR

D) Gating for CD163 positivity on singlet population using FMO for CD163

Supplementary Figure 3

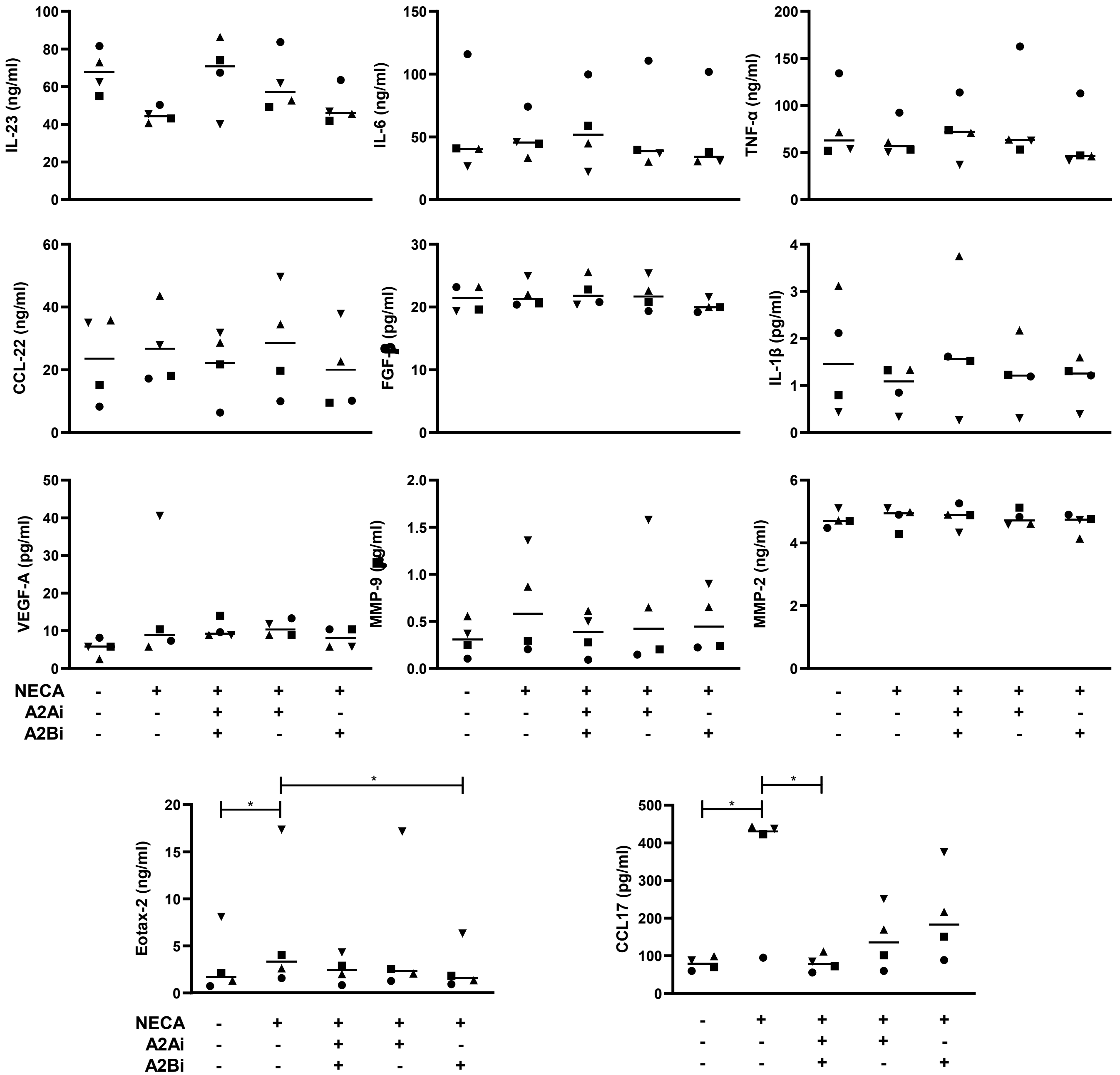

GM-CSF macrophages conditioned with NECA (1µM) +/- A2A inhibitor (SCH442416, 1µM) and/or A2B inhibitor (PSB603, 1µM) were stimulated with LPS/IFNγ for 24hrs and supernatants collected for Luminex analysis. Raw data for singlicate determination for each of 4 donors with line at median.

Supplementary Figure 4

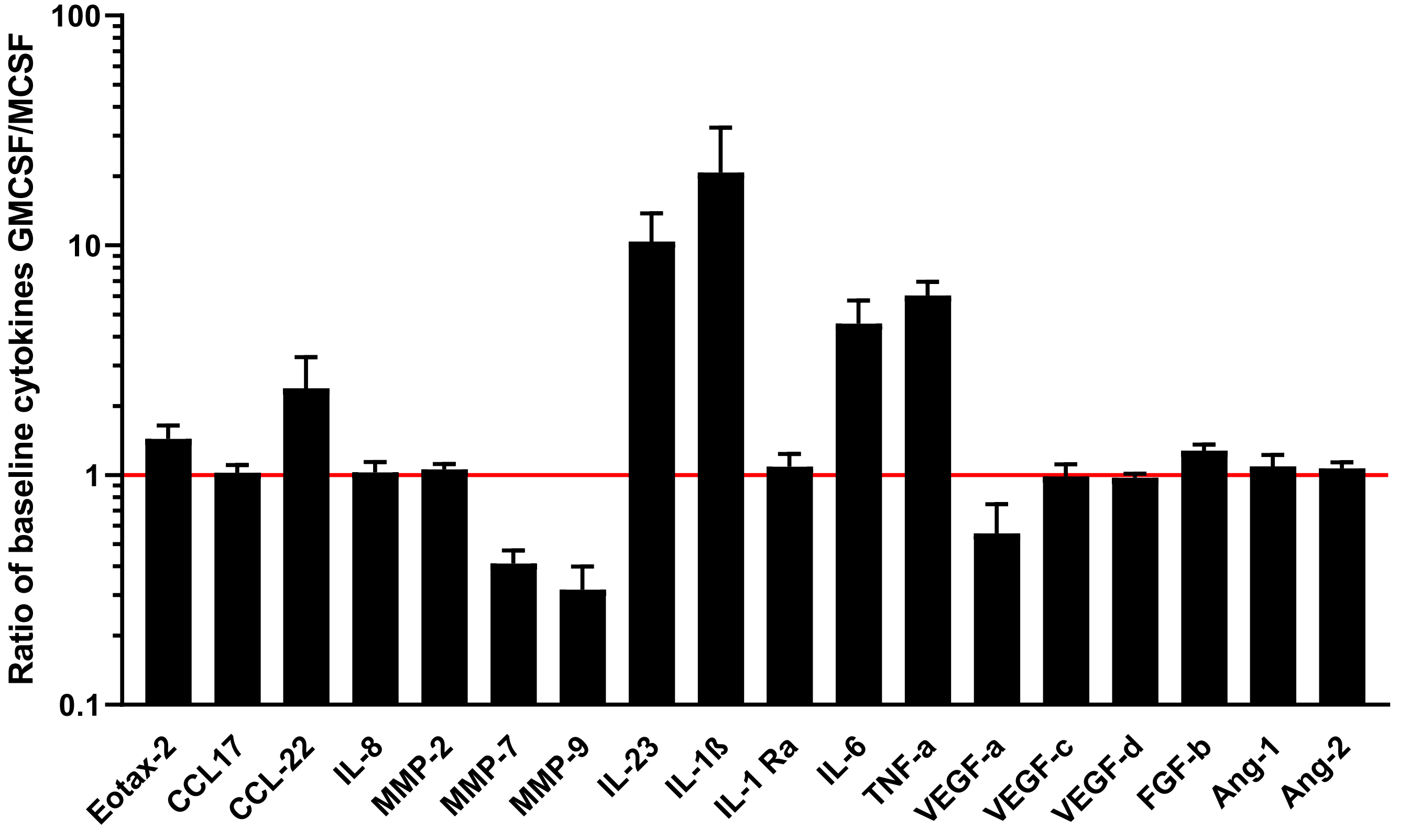

GM- Mϕ and M- Mϕ were stimulated with LPS/IFNγ or LPS respectively for 24hrs and supernatants collected for Luminex analysis. Shown is mean +/- SEM of ratio of cytokines between GM- Mϕ and M- Mϕ from 4 donors. Red line at ratio of 1 indicates equivalent secretion by both macrophage types.

Supplementary fig 5

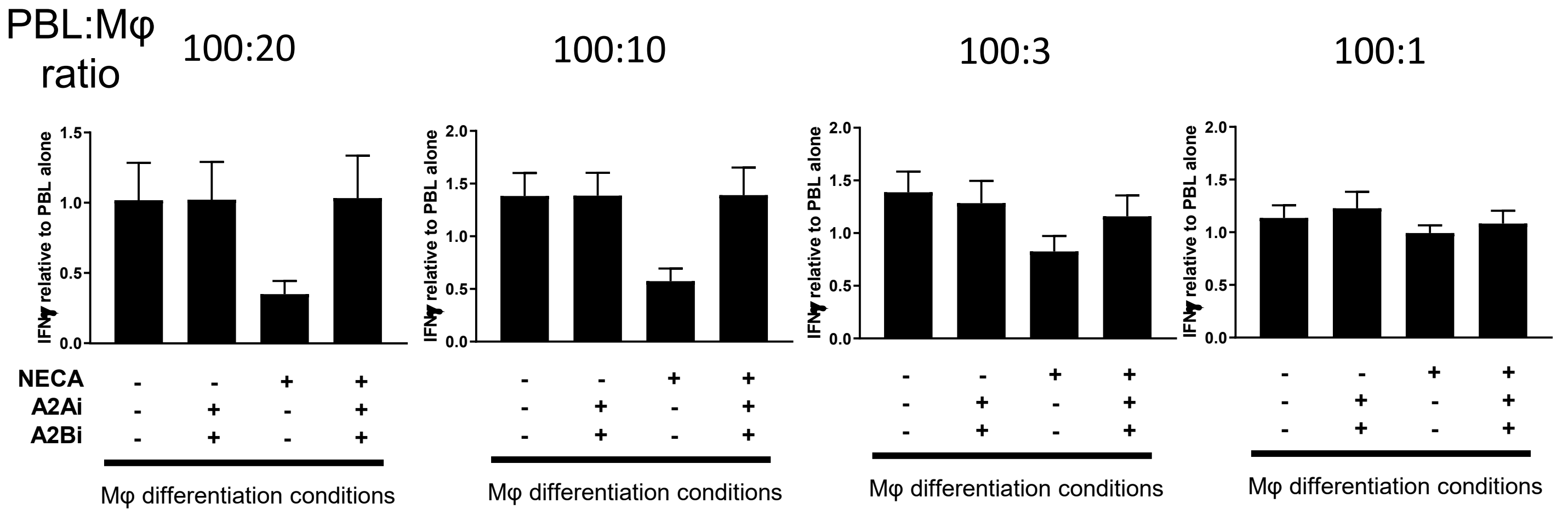

IFNγ measured by ELISA in supernatants after 4 days of co-culture of PBL + PHA with M-CSF macrophages conditioned with A2A/A2B inhibitors, NECA or both. Shown is IFNγ relative to secretion by PBL stimulated with PHA in absence of macrophages.
